# Amino acid 159 of the envelope protein affects viral replication and T-cell infiltration by West Nile virus in intracranial infection

**DOI:** 10.1038/s41598-020-64199-7

**Published:** 2020-04-28

**Authors:** Shintaro Kobayashi, Chisato Kaneko, Ryoko Kawakami, Rie Hasebe, Hirofumi Sawa, Kentaro Yoshii, Hiroaki Kariwa

**Affiliations:** 1 Laboratory of Public Health, Faculty of Veterinary Medicine, Hokkaido University, N18, W9, Kita-ku Sapporo, 060-0818 Japan; 2 Biomedical Animal Research Laboratory, Institute for Genetic Medicine, Hokkaido University, N15, W7, Kita-ku Sapporo, 060-0815 Japan; 3 Division of Molecular Pathobiology, Research Center for Zoonosis Control, Hokkaido University, N20, W10, Kita-ku Sapporo, 001-0020 Japan; 4 Global Institution for Collaborative Research and Education (GI-CoRE), Hokkaido University, Sapporo, Japan; 5 Global Virus Network, Baltimore, MD USA

**Keywords:** Viral pathogenesis, West nile virus

## Abstract

West Nile virus (WNV) is an important cause of viral encephalitis in birds and animals, including humans. Amino acid 159 of the envelope (E) protein is reportedly implicated in the different levels of neurovirulence in mice infected with WNV NY99 or Eg101. We investigated the role of amino acid 159 of the E protein in the pathogenesis of WNV infection. We produced recombinant WNV with the structural proteins of the NY99 or Eg101 strain (NY-WT or EgCME-WT) and mutant viruses with substitutions of amino acid 159 of the E protein (NY-E-V159I or EgCME-E-I159V). The NY-WT and NY-E-V159I or EgCME-WT and EgCME-E-I159V titers in culture supernatant were similar. The mortality rate and viral titer in the brains of mice inoculated intraperitoneally with NY-WT or NY-E-V159I were also similar. In contrast, the mortality rate and viral titer in the brains of mice inoculated intracranially with EgCME-E-I159V were significantly higher than those of mice inoculated with EgCME-WT. The numbers of CD3-positive and CD8-positive T cells were greater in brains inoculated with EgCME-E-I159V than in those inoculated with EgCME-WT. Therefore, amino acid 159 of the E protein modulates the pathogenicity of WNV by affecting viral replication and T-cell infiltration in the brain.

## Introduction

West Nile virus (WNV) is a positive-sense RNA flavivirus classified phylogenetically into five distinct genetic lineages^1–3^. In nature, WNV is maintained in a transmission cycle between mosquitoes and birds. WNV infection in dead-end hosts (*e*.*g*. human or horse) results in outcomes ranging from febrile to severe neurological disease, including meningitis, encephalitis, and death^4^. Although WNV vaccines for horses have been developed^5^, no vaccine or therapy for WNV is currently approved for human use.

The WNV genome encodes a single polyprotein, which is cleaved by cellular and viral proteases into three structural proteins—capsid, pre-membrane (prM), and envelope (E)—and seven nonstructural (NS) proteins—NS1, NS2A, NS2B, NS3, NS4A, NS4B, and NS5. WNV virions are approximately 50 nm in diameter and have an outer shell comprising the M and E proteins^6^. The E protein is responsible for entry of WNV into cells and is an important target of neutralising antibodies^7^. The crystal structure of the E protein reveals three distinct domains: a β-barrel–shaped domain I, an elongated finger-like domain II, and a C-terminal immunoglobulin-like domain III^8^. Domain I, in the center of the E protein, has a glycosylated amino acid at position 154, which is important for WNV infection of vertebrates^9^. The internal fusion peptide loop at the tip of domain II promotes the trimerisation of the E protein for the initiation of viral entry^8^. Domain III mediates the attachment of WNV to host cells and is targeted by a number of neutralising monoclonal antibodies^8^.

Strains representative of genetic lineages 1 and 2 are found to have a wide spectrum of virulence phenotypes ranging from severe to mild^10^. The WNV strains isolated in New York City in 1999 (NY99) were more neuroinvasive than other lineage-1 strains, such as that from Egypt (Eg101), in a mouse model^11^. Comparison of the NY99 and Eg101 strains suggested roles for amino acids 156 and 159 of the E protein as determinants of neuroinvasion^12^. These two amino acids play roles in intracellular transport in endothelial cells *in vitro*^12^. Amino acid 156 is involved in glycosylation of the E protein, which is implicated in virion stability and neurovirulence *in vivo*^10^. However, the role of amino acid 159 in the neuropathogenesis of WNV is poorly understood.

In this study, we examined the role of amino acid 159 of the E protein in the pathogenesis of WNV in a mouse model by creating mutant WNV strains.

## Results

### Effects of amino acid 159 of the E protein on viral replication and the properties of the E protein

**Figure 1:**
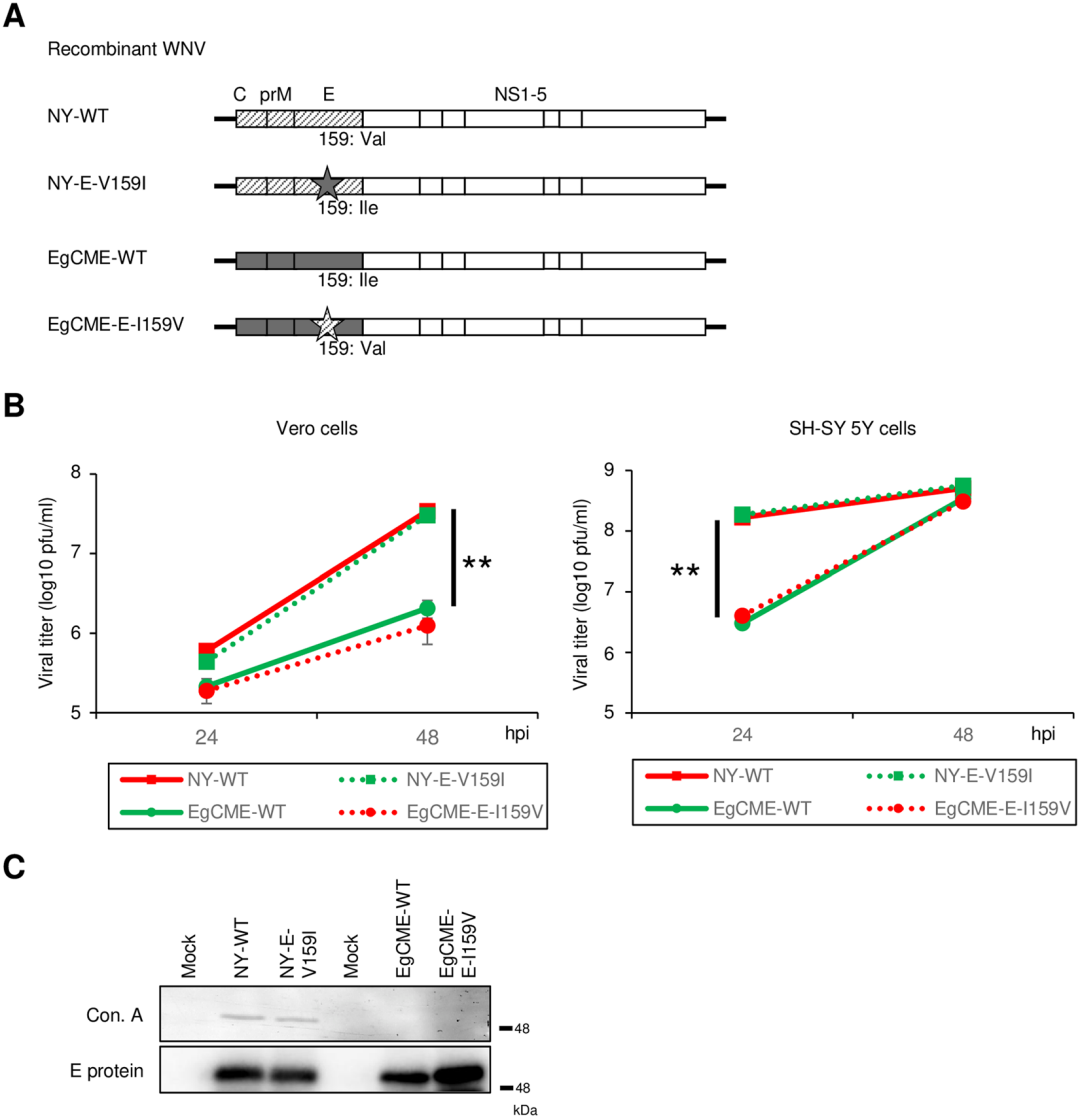
Characterisation of recombinant WNV. (**A**) Schematic of the genome of recombinant WNV. Diagonal lines and grey boxes, structural proteins of NY99 and Eg101, respectively; star, amino acid substitution at position 159 of the E protein. (**B**) Growth of NY-WT, NY-E-V159I, EgCME-WT, and EgCME-E-I159V. Vero or SH-SY 5Y cells were inoculated with recombinant WNV, culture supernatants were harvested, and viral titers were determined by plaque assay. Data are means ± standard errors from three independent experiments. Statistical significance was assessed by the two-tailed Student’s t-test. **p < 0.01. (**C**) Analysis of E protein glycosylation in recombinant WNV. Vero cells were infected with WNV, and intracellular E protein was immunoprecipitated using an anti-E protein antibody. Precipitated E protein was detected using concanavalin A (ConA) or an anti-E protein antibody. Cropped blots are shown; full-length blots are presented in Supplementary Fig. S2.

Recombinant wild-type NY99 (NY-WT) virus was recovered by homologous recombination as reported previously^13^. Chimeric WNV (EgCME-WT) was generated by replacing the sequence encoding the structural proteins of NY99 with that of Eg101. To investigate the role of amino acid 159 of the E protein in pathogenesis, Val-to-Ile and Ile-to-Val mutations were introduced into NY-WT and EgCME-WT, respectively (NY-E-V159I and EgCME-E-I159V; Fig. 1A).

To examine whether those mutations affected viral multiplication, Vero and SH-SY 5Y cells were inoculated with the recombinant viruses. Although the viral titer of EgCME-WT was significantly lower than that of NY-WTs, the titer of NY-E-V159I or EgCME-E-I159V was similar to that of NY-WT or EgCME-WT at each time point in both cell lines, respectively (Fig. 1B). Therefore, mutation of amino acid 159 of the E protein did not affect viral multiplication in cell culture.

N-linked glycosylation of amino acid 154 of the E protein is reportedly important for the release of viral particles and neuroinvasion in mice^10,14^, and is affected by neighbouring amino acids^12,15^. To examine the effect of mutation on the glycosylation of the E protein, Vero cells were infected with the recombinant viruses, and intracellular E protein was immunoprecipitated using an anti-E protein antibody and analysed by lectin blotting. The E protein was detected in the precipitate from cells infected with each virus strain (Fig. 1C, lower panel). The E protein of NY-WT and NY-E-V159I was detected by ConA, which binds specifically to high-mannose type N-linked glycans, whereas that of EgCME-WT and EgCME-E-I159V was not detected by ConA (Fig. 1C, upper panel). These results indicated that mutation of position 159 in the E protein did not affect its glycosylation.

**Figure 2:**
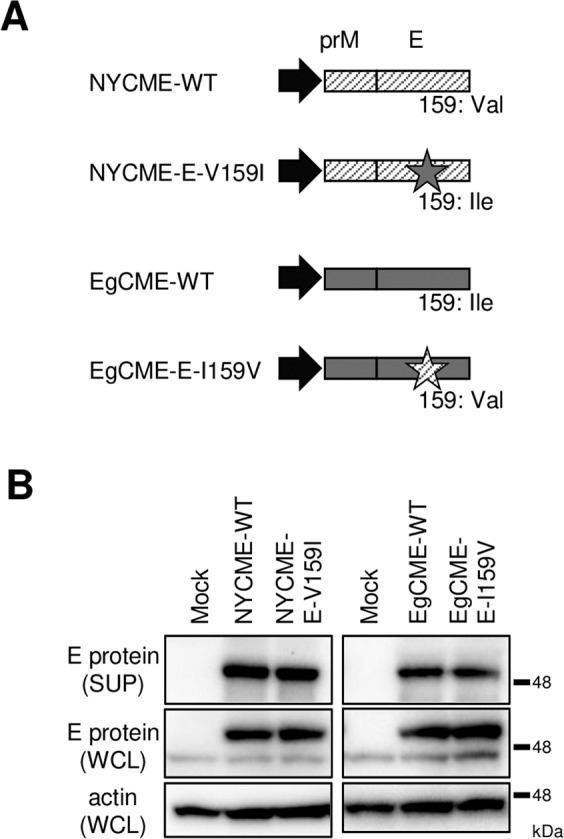
Effect of mutation on the release of viral particles. (**A**) Schematic of the plasmids harboring genes encoding the prM and E proteins. Diagonal lines and grey boxes, structural proteins of NY99 and Eg101, respectively; star, amino acid substitution at position 159 of the E protein; black arrow, CMV promoter region. (**B**) Release of viral particles. Culture supernatants (SUP) and whole-cell lysates (WCL) from cells expressing the E protein were analysed by immunoblotting for E protein and actin. Cropped blots are shown; full-length blots are presented in Supplementary Fig. S3.

Recombinant subviral particles (SPs) are formed in cells producing only prM and E proteins, and are secreted similarly to authentic virions^16^. SPs are useful for assessment of the assembly and secretion of WNV particles independent of virion entry and viral genome replication^16,17^. The prM and E proteins of NY99 or Eg101 with/without a substitution at position 159 of the E protein were produced in 293 T cells (Fig. 2A), and the intracellular and extracellular E protein levels were analysed by immunoblotting. The extracellular and intracellular E protein levels were not affected by mutation at position 159 (Fig. 2B). Therefore, mutation of amino acid 159 did not affect the release of viral particles.

In summary, mutation of amino acid 159 of the E protein did not affect viral replication, glycosylation of the E protein, or particle release.

### Effects of amino acid 159 of the E protein on the neuroinvasion and neurovirulence of WNV

**Figure 3:**
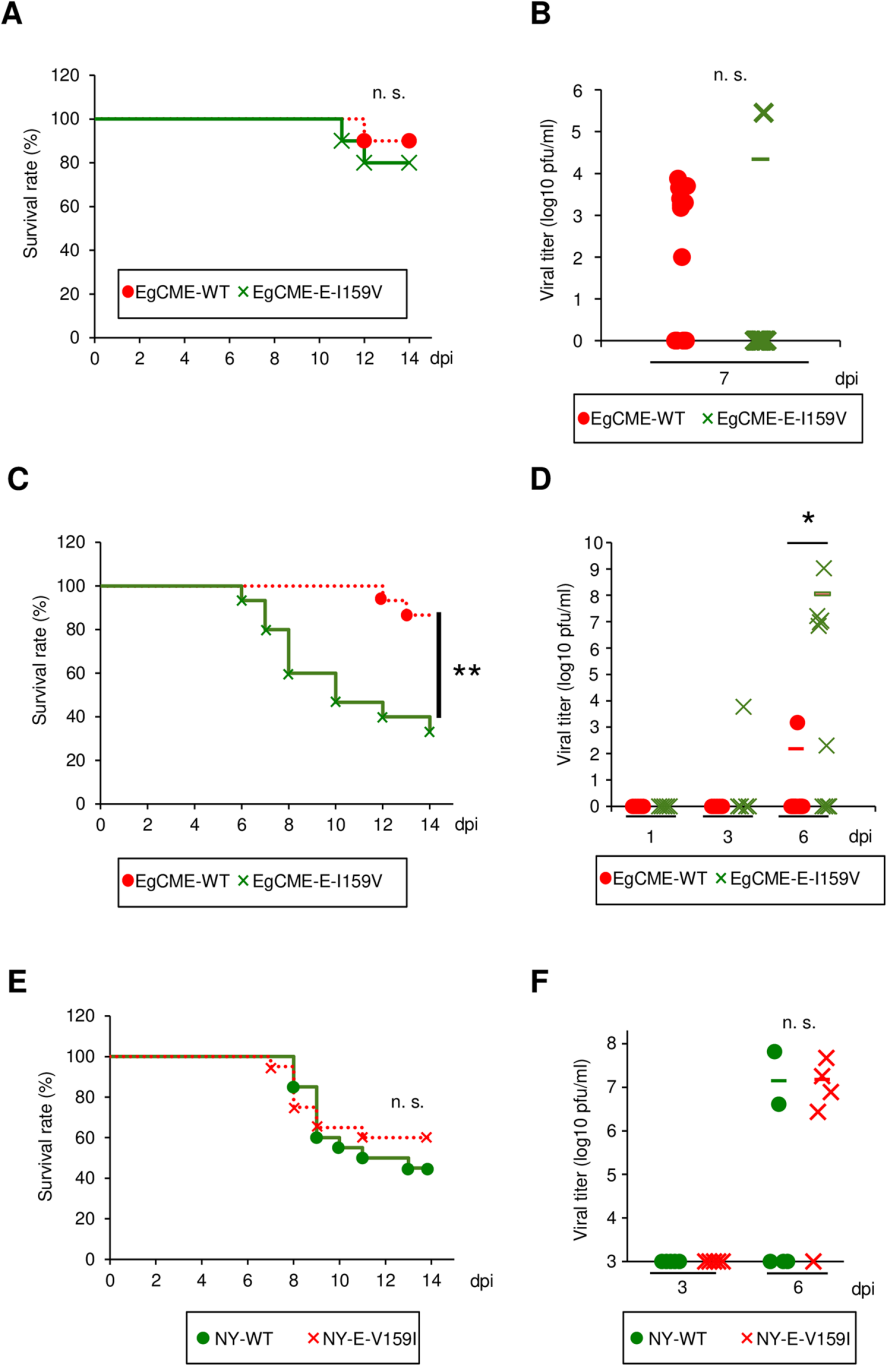
Analysis of mice inoculated with recombinant WNV. C57BL/6 mice were inoculated intraperitoneally with 10,000 pfu EgCME-WT or EgCME-E-I159V (A and B), intracranially with 10 pfu NY-EgCME or NY-EgCME-E159V (C and D), or intracranially with 1 pfu NY-WT or NY-E-V159I (E and F). (**A**) Kaplan-Meier survival curves of mice inoculated intraperitoneally with EgCME-WT or EgCME-E-I159V (*n* = 10). n.s., not significant. (**B**) Brains were collected at 7 dpi and the viral titers were measured (n = 13). Statistical significance was assessed by the two-tailed Student’s *t*-test. n.s., not significant. (**C**) Kaplan-Meier survival curves of mice inoculated intracranially with EgCME-WT or EgCME-E-I159V (*n* = 15). **p < 0.01. (**D**) Brains were collected at 1, 3, and 6 dpi and the viral titers were measured. Statistical significance was assessed by the two-tailed Student’s *t*-test. *p < 0.05. (**E**) Kaplan-Meier survival curves of mice inoculated intracranially with NY-WT or NY-159I (*n* = 20). n.s., not sig*n*ificant. (**F**) The brains were collected at 3 or 6 dpi and the viral titers were measured. Statistical significance was assessed by the two-tailed Student’s *t*-test. n.s., not significant.

To examine the roles of amino acid 159 in neuroinvasion and neurovirulence, recombinant viruses were inoculated intraperitoneally or intracranially into C57BL/6 J mice. The survival rate after intraperitoneal inoculation (10,000 pfu/mouse) of EgCME-E-I159V was 80%, similar to that following inoculation of EgCME-WT (90%; Fig. 3A). No significant difference in the viral titer was detected in brains inoculated with each of the virus strains at 7 dpi (Fig. 3B).

After intracranial inoculation (10 pfu/mouse), the survival rate of mice inoculated with EgCME-E-I159V was significantly lower than that of those inoculated with EgCME-WT (Fig. 3C). The EgCME-E-I159V titer in the brain was significantly higher than that of EgCME-WT at 6 dpi (Fig. 3D). The survival rate of mice inoculated intracranially (1 pfu/mouse) with NY-E-V159I was non-significantly higher than that of those that received NY-WT (Fig. 3E). The viral titer in the brain did not differ significantly between NY-WT and NY-E-V159I (Fig. 3F).

Taken together, the above results indicated that amino acid 159 of the E protein influences viral replication and pathogenesis in the brain but does not affect neuroinvasion.

### Effect of amino acid 159 of the E protein on the pathogenicity of WNV

**Figure 4:**
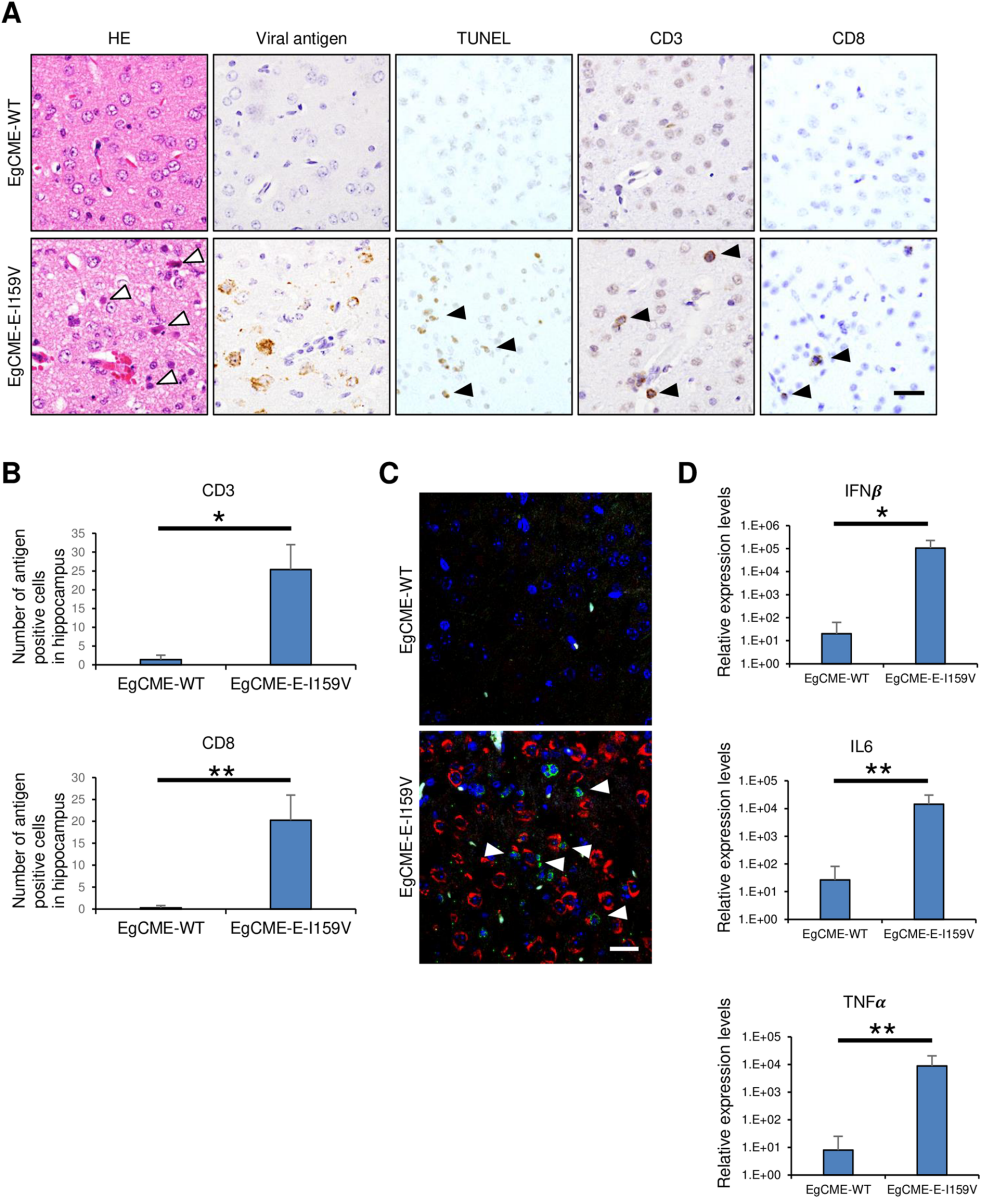
Infiltration of T cells in mouse brains infected with recombinant WNV. C57BL/6 mice were inoculated intracranially with 10 pfu EgCME-WT or EgCME-E-I159V. (**A**) Images of haematoxylin and eosin-stained sections of the cerebral cortex, sections stained by TUNEL or immunostained for WNV antigen, CD3, or CD8 (*n* = 5). White arrowheads, degenerated neuronal cells; black arrowheads, TUNEL-positive cells, CD3-positive cells, or CD8-positive cells. Scale bar, 25 μm. (**B**) Number of CD3- or CD8-positive cells in the hippocampus (n = 5). Statistical significance was assessed by the two-tailed Student’s *t*-test. *p < 0.05; **p < 0.01. (**C**) Relationship between CD3-positive T cells and WNV antigen-positive cells. Sections were stained with antibodies against CD3 (green) and WNV antigen (red). Nuclei were stained with DAPI (blue). White arrowheads indicate CD3-positive T cells adjacent to viral antigen-positive cells. Scale bar, 20 μm. (**D**) Expression levels of genes encoding immune factors in brains inoculated with EgCME-WT or EgCME-E-I159V. Harvested brains were subjected to RT-qPCR for IFNβ, IL6 and TNFα (n = 5). Statistical significance was assessed by the Man-Whitney’s *U*-test. *p < 0.05; **p < 0.01.

Infiltration of T cells in the brain is important for the pathogenesis of flavivirus infection in mice^18–20^. To assess the role of amino acid 159 of the E protein in the pathogenesis, we examined the infiltration of T cells in brains infected with recombinant WNV. In brains inoculated with EgCME-E-I159V, many degenerated cells with eosinophilic cytoplasm were observed (Fig. 4A, white arrowheads), together with viral antigen-positive cells (Fig. 4A). TUNEL-positive cells were also detected in the cerebral cortex region of brains inoculated with EgCME-E-I159V (Fig. 4A), indicating that EgCME-E-I159V induced cell death. In contrast, these pathological changes were rare in brains inoculated with EgCME-WT (Fig. 4A). In brains inoculated with EgCME-E-I159V, CD3 (T-cell marker)- or CD8 (cytotoxic T-cell marker)-positive T cells were detected (Fig. 4A), the numbers of which were significantly greater than in brains inoculated with EgCME-WT (Fig. 4B). Infiltration of CD3- or CD8-positive T cells was also examined in brains inoculated intracranially with NY-WT or NY-E-V159I. No difference in the infiltration of CD3- or CD8-positive T cells was observed in brains inoculated intracranially with NY-WT or NY-E-V159I (Supplementary Fig. S1). To examine whether T-cell infiltration was affected by WNV infection, localisation of CD3-positive T cells and WNV antigen-positive cells in brain was analysed by immunohistochemistry. Many CD3-positive T cells adjacent to viral antigen-positive cells were observed in the brain inoculated with EgCME-E-I159V, but not in that inoculated with EgCME-WT (Fig. 4C). Furthermore, EgCME-E-I159V infection significantly increased the expression levels of the genes encoding interferon (IFNβ) and factors related to antibody and inflammatory responses (IL6 and TNFα, respectively) as compared with EgCME-WT infection (Fig. 4D). These results indicated that amino acid 159 of the E protein is involved in WNV infection and T-cell infiltration of the brain.

## Discussion

In this study, the role of amino acid 159 of the E protein in the pathogenesis of WNV in mice was investigated using mutant recombinant WNV produced by reverse genetics. Amino acid 159 of the E protein played roles in viral multiplication and pathogenicity in the brain, but did not affect viral glycosylation or multiplication in cultured cells. Mutation of amino acid 159 affected neuronal cell death and T-cell responses in the brain. Therefore, amino acid 159 of the E protein is likely associated with the induction of an antiviral immune response.

The immune system is important in WNV infection^19–21^. Several immune factors—inflammatory cytokines, chemokines, complement, B cells, and T cells—are important for viral replication and disease^19,20,22^. In this study, mutation of amino acid 159 of the E protein affected the infiltration of CD3- and CD8-positive T cells in the brain. Aberrant T-cell infiltration in brain was reported to be linked to the pathogenesis of WNV infection^23^. Amino acid 159 of the E protein may thus modulate T-cell infiltration and the immunopathogenic/immunoprotective balance in the brain during WNV infection.

Mutation of amino acid 159 of the E protein also modulated the rate of brain cell death. Infection by flaviviruses, including WNV, induces ER stress and the unfolded protein response (UPR) to support viral growth and modulate apoptotic signalling^24–26^. During infection with dengue virus, the E protein interacts with chaperone proteins involved in ER stress and the UPR in the ER^27^. Therefore, amino acid 159 of the E protein may suppress the function of chaperone proteins, causing cell death by inducing ER stress.

The mechanism of WNV invasion from peripheral tissues to the central nervous system is unclear. Several modes of passage across the blood–brain barrier have been suggested: a transcellular pathway mediated by vesicles, paracellular entry after disruption of tight junctions, and a Trojan-horse mechanism by transport within circulating phagocytic cells^12,28^. The transcellular transport in endothelial cells of viral particles containing the NY99-type (Val) amino acid at position 159 of the E protein was greater than that of particles with the Eg101-type (Ile) amino acid^12^. In this study, substitution of amino acid 159 in the E protein of Eg101 from Ile to Val did not affect the survival rate or viral multiplication in the brains of mice inoculated intraperitoneally. Thus, transcellular activity may not be important for the neuroinvasiveness of WNV in mice.

The neuropathogenicity of NY99 in mice was not affected by mutation of the E protein at position 159. NY99 is reportedly more virulent than Eg101^11^, and various viral factors are implicated in the pathogenicity of WNV. The C and NS2B/3 proteins are important for the induction of apoptosis and inflammation^29,30^, and the NS5 protein—an antagonist of type-I interferon signalling—is related to the virulence of WNV^31^. Therefore, amino acid substitution at position 159 may not attenuate the virulence of the highly virulent NY99.

The glycosylation of E protein mediates the virulence of WNV in a mouse model^10^. The glycosylation varies among WNV strains, and it was reported that the E protein of the Eg101 strain was not glycosylated, unlike that of the NY99 strain^12^. Glycosylation is reportedly affected by amino acid 156, which differs between NY99 and Eg101; by contrast, amino acid 159 is unrelated to glycosylation^12^. In this study, substitution of amino acid 159 in the E protein of the Eg101 strain did not affect the glycosylation but increased virulence in a mouse model. These results suggested that increase in virulence caused by that substitution was independent of the glycosylation.

In summary, amino acid 159 of the E protein is a determinant of the neurovirulence of WNV and affects host responses, including the immune response, related to neuronal cell degeneration. However, the detailed mechanisms are unclear, and further work is needed. Elucidation of the host response to amino acid 159 will improve our understanding of the molecular pathogenicity of WNV infection and enable the identification of potential therapeutic targets for West Nile encephalitis.

## Methods

### Cells

Vero cells, obtained from the JCRB Cell Bank (JCRB0111), were cultured in an atmosphere containing 5% CO_2_ at 37 °C in modified Eagles’ medium (MEM; Wako, Osaka, Japan) supplemented with 10% heat-inactivated fetal bovine serum (FBS). SH-SY 5Y cells, obtained from the European Collection of Authenticated Cell Cultures (94030304), were cultured in an atmosphere containing 5% CO_2_ at 37 °C in Dulbecco’s MEM (DMEM)/Nutrient Mixture F-12 Ham (Wako) supplemented with 10% heat-inactivated FBS. HEK-293T cells, kindly provided by Dr. Matsuura (Osaka University), were cultured in an atmosphere containing 5% CO_2_ at 37 °C in high-glucose DMEM (Wako) supplemented with 10% heat-inactivated FBS.

### Viruses

The WNV 6-LP strain was established as described previously^14^. The Eg101 strain of WNV was provided by Dr. Duane Gubler of the Centers for Disease Control and Prevention (Fort Collins, CO, USA). Experiments involving infectious WNV were performed in the Biosafety Level-3 (BSL-3) facility of Hokkaido University, Japan, according to the institutional guidelines.

### Antibodies

Rabbit anti-JEV serum that exhibited cross-reactivity with the WNV E protein was prepared as described previously^32–34^. The following antibodies were used: mouse anti-WNV E protein monoclonal antibody (Merck Millipore, Billerica, MA, USA), mouse anti-actin monoclonal antibody (Wako), rabbit anti-CD3E monoclonal antibody (Sigma-Aldrich, St. Louis, MO, USA), and rabbit anti-CD8a monoclonal antibody (Cell Signaling Technology, Beverly, MA, USA).

### Construction of plasmids

To produce recombinant WNV, the region encoding the structural proteins of WNV 6-LP and Eg101 was cloned into pCR-2.1 (Thermo Fisher Scientific, Waltham, MA, USA), resulting in pCR-NY99CME and pCR-Eg101CME, respectively. A plasmid harboring subgenomic replicon RNA, which lacks most of the sequence encoding the structural proteins (pCMV-WNrep-DsRed), was constructed previously^13^.

To produce recombinant subviral particles, the sequences encoding the prM and E proteins of WNV 6-LP or Eg101 were cloned into the pCXSN plasmid, which was generated from the pCMV-Myc plasmid (TaKaRa Bio USA, Inc., Mountain View, CA, USA). Mutations were introduced into the sequences encoding the structural proteins by inverse PCR.

### Production of recombinant WNV

Recombinant WNV was produced as described previously^13^. Briefly, purified DNA fragments encoding the structural proteins and pCMV-WNrep-DsRed were transfected into HEK-293T cells. After 3 days, the supernatant was collected and inoculated onto Vero cells for 5 days; thereafter, the supernatant was collected.

### Growth assay and titration

Subconfluent Vero cells or SH-SY 5Y cells were inoculated with WNV at 0.001 or 1 plaque-forming unit (pfu)/cell, respectively, and cultured for 24, 48, or 72 h. The supernatants of WNV-infected cell cultures were collected and stored at −80 °C until determination of viral titer. The viral titer was measured as described previously^13^.

### Immunoprecipitation, immunoblotting, and lectin blotting

Cell lysates were prepared as described previously^34^. The lysates were precleared on SureBeads Protein G (Bio-Rad, Hercules, CA) for 30 min at 4 °C. The precleared lysates were precipitated using SureBeads Protein G and a mouse monoclonal anti-E antibody (Merck Millipore) for 3 h at 4 °C. Immunocomplexes were electrophoresed through sodium dodecyl sulphate polyacrylamide gels and transferred to a polyvinylidene difluoride membranes (Merck Millipore). The membrane was blocked with 5% skim milk in Tris-buffered saline with Tween, and incubated overnight with the indicated antibodies or biotinylated lectin concanavalin A (ConA; J-Oil Mills) at 4 °C. The complexes were detected using horseradish peroxidase (HRP)-conjugated secondary antibodies or alkaline phosphatase-conjugated streptavidin (Jackson ImmunoResearch). Protein bands were visualised as described previously^34^.

### Transfection and ultracentrifugation

pCXSN-prMENY99 or -prMEEg101 was transfected into HEK293T cells using the X-tremeGENE HP DNA Transfection Reagent (Roche, Basel, Switzerland), following the manufacturer’s instructions. The cells were incubated for 48 h at 37 °C, and the cells and culture supernatants were harvested and stored at −80 °C. The supernatants (containing E protein) were concentrated by ultracentrifugation as described previously^35^.

### Ethics statement

All animal experiments were performed at the Animal BSL-3 facility of the Graduate School of Veterinary Medicine of Hokkaido University, which has been certified by The Association for Assessment and Accreditation of Laboratory Animal Care International, and followed the basic guidelines for animal experiments of the Ministry of Education, Culture, Sports, Science, and Technology (MEXT) of Japan. All animal experiments were approved by the President of Hokkaido University after review by the Animal Care and Use Committee of Hokkaido University (approval no. 13025).

### Inoculation of mice with WNV

Six-week-old C57BL/6JJmsSlc mice were obtained from Japan SLC Inc. (Shizuoka, Japan). The mice were anesthetised using sevoflurane and infected with WNV by intraperitoneal or intracranial inoculation. After euthanasia at 1, 3, 6, or 7 days post-inoculation (dpi), the brains of the mice were collected.

### Analysis of tissue samples

For virological analysis, the tissue samples were weighed, homogenised as 10% suspensions (wt/vol) in PBS, and quantitated by plaque assay as described above.

Pathological analysis was performed as described previously^33^. For immunohistochemical analysis, deparaffinised sections were subjected to antigen retrieval by trypsin digestion at 37 °C for 10 min for the WNV antigen and by heating in a pressure cooker in 0.01 M citrate buffer (pH 6.0) for CD3 or CD8, and were immunostained as described previously^33^. For quantification of CD3 or CD8-positive cells, the antigen-positive cells in the hippocampus were counted and calculated as described previously^34^. For detection of apoptotic cells, an *in situ* cell death detection kit (Roche) was used according to the manufacturer’s instructions.

### Quantitative reverse-transcriptase polymerase chain reaction

Total RNA was isolated from the brains of WNV-inoculated mice using Isogen II (Nippon Gene) according to the manufacturer’s protocol. Complementary DNA synthesis was performed with random primers using SuperScript III Reverse Transcriptase (Thermo Fisher Scientific). Quantitative PCR was performed with the KAPA SYBR Fast qPCR Kit (Kapa Biosystems, Wilmington, MA, USA) and the 7500 Fast Real-Time PCR System (Thermo Fisher Scientific). The primers used were as follows (forward and reverse): 5′-GGAGATGACGGAGAAGATGC-3′ and 5′-CCCAGTGCTGGAGAAATTGT-3′ for IFNβ, 5′-GTTCTCTGGGAAATCGTGGA-3′ and 5′-TGTACTCCAGGTAGCTATGG-3′ for IL6, and 5′-GCACAGAAAGCATGACCCG-3′ and 5′-GCCCCCCATCTTTTGGG-3′ for TNFα.

## Supplementary information

Supplementary figures.
