# Supplementary material for: Amino acid 159 of the envelope protein affects viral replication and T-cell infiltration by West Nile virus in intracranial infection
Source: Sci Rep. 2020 Apr 28;10:7168. doi: 10.1038/s41598-020-64199-7 (PMC7189269; doi:10.1038/s41598-020-64199-7)
Supplement: Supplementary file 1 — Supplementary figures. [file 41598_2020_64199_MOESM1_ESM.pdf]

Amino acid 159 of the envelope protein affects viral replication and T-cell infiltration by West Nile virus in intracranial infection

Shintaro Kobayashi[1*], Chisato Kaneko[1], Ryoko Kawakami[1], Rie Hasebe[2], Hirofumi Sawa[3, 4, 5], Kentaro Yoshii[1], Hiroaki Kariwa[1]

1. Laboratory of Public Health, Faculty of Veterinary Medicine, Hokkaido University, N18, W9, Kita-ku, Sapporo, 060-0818, Japan
2. Biomedical Animal Research Laboratory, Institute for Genetic Medicine, Hokkaido University, N15, W7, Kita-ku, Sapporo, 060-0815, Japan
3. Division of Molecular Pathobiology, Research Center for Zoonosis Control, Hokkaido University, N20, W10, Kita-ku, Sapporo, 001-0020, Japan
4. Global Institution for Collaborative Research and Education (GI-CoRE), Hokkaido University, Sapporo, Japan
5. Global Virus Network, Baltimore, MD, USA

Supplementary Information

Supplementary Figure S1

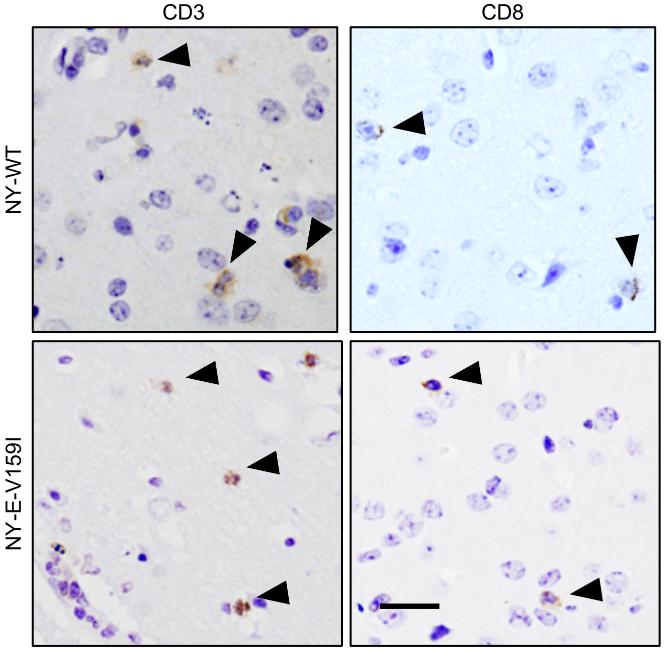

Supplementary Figure S1: Images of sections of the cerebral cortex. C57BL/6 mice were inoculated intracranially with 1 pfu NY-WT or NY-E-V159I. The sections were immunostained for CD3 or CD8. Black arrowheads, CD3-positive cells or CD8-positive cells. Scale bar, 20 μm.

Supplementary Figure S2

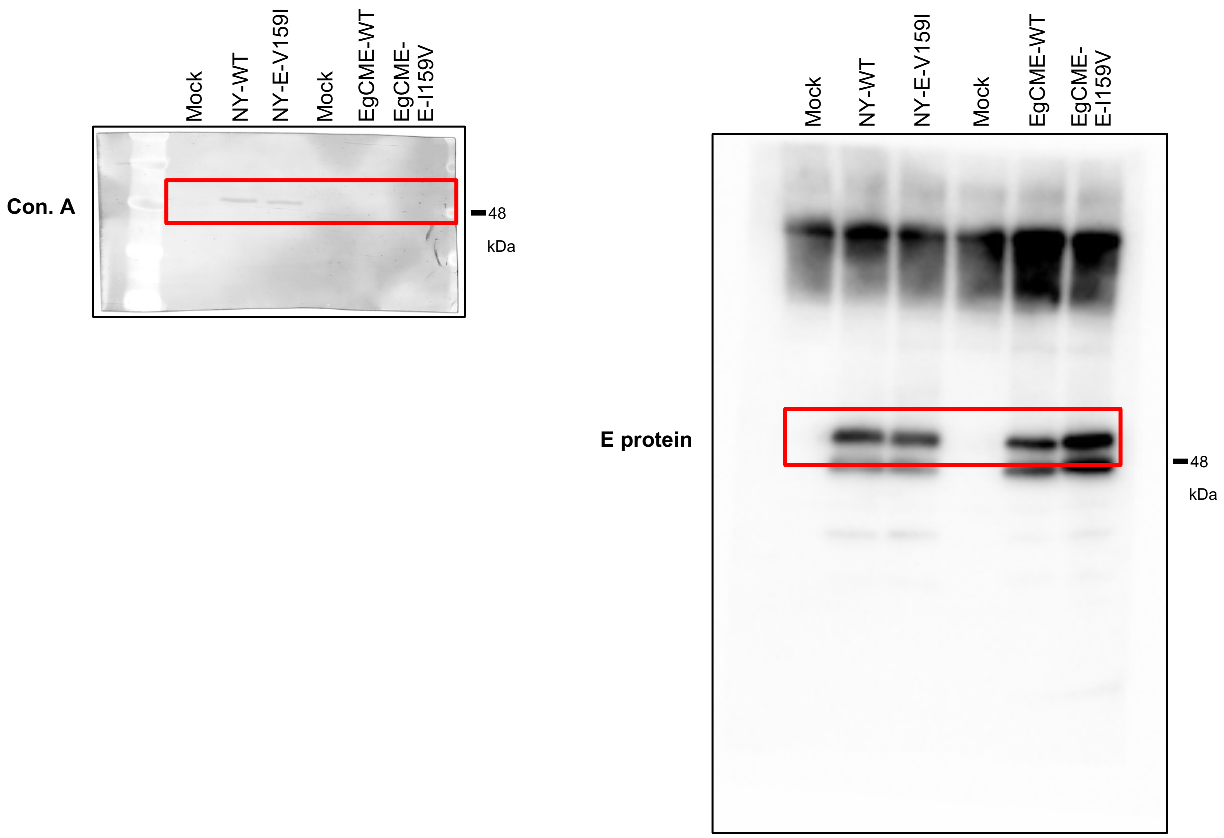

Supplementary Figure S2: Full-length blots of Figure 1C. Red-boxed regions were shown in Fig. 1C

Supplementary Figure S3

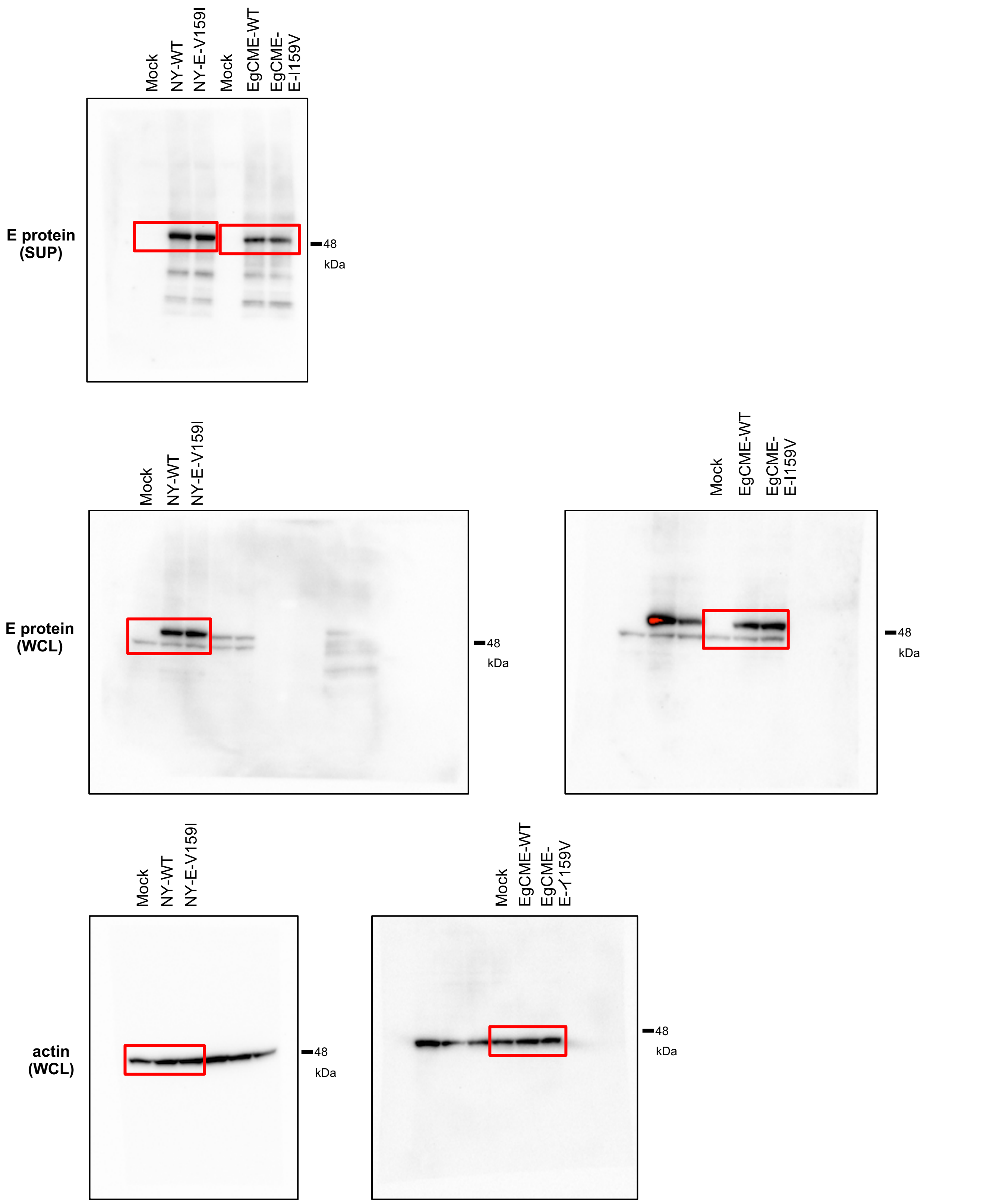

Supplementary Figure S3: Full-length blots of Figure 2B. Red-boxed regions were shown in Fig. 2B
